# A113 FACILITATORS FOR EVIDENCE-BASED INTERVENTIONS FOR PSYCHOLOGICAL DISTRESS IN PERSONS LIVING WITH IBS: A ROADMAP FOR HUMAN-CENTERED DESIGN AND IMPLENTATION

**DOI:** 10.1093/jcag/gwae059.113

**Published:** 2025-02-10

**Authors:** M C MacDonald, C Heisler, N Willett, N Rohatinsky, S Farina, M Stewart, M Vallis, T Shepherd, B Currie, J Robar, T Huard, E Neil, J Jones

**Affiliations:** Dalhousie University, Halifax, NS, Canada; Dalhousie University, Halifax, NS, Canada; Dalhousie University, Halifax, NS, Canada; Dalhousie University, Halifax, NS, Canada; Dalhousie University, Halifax, NS, Canada; Dalhousie University, Halifax, NS, Canada; Dalhousie University, Halifax, NS, Canada; Dalhousie University, Halifax, NS, Canada; Dalhousie University, Halifax, NS, Canada; Dalhousie University, Halifax, NS, Canada; Dalhousie University, Halifax, NS, Canada; Dalhousie University, Halifax, NS, Canada; Dalhousie University, Halifax, NS, Canada

## Abstract

**Background:**

IBD-related psychological distress (IBD-PD) refers to the emotional impact of IBD and has been associated with increased disease severity, co-morbid mental health disorders (i.e., anxiety and depression), and increased mortality. Ensuring high-quality, patient-centered care for IBD-PD that is proportionate to the level of distress severity will address a key evidence-practice gap in our healthcare system.

**Aims:**

This study aims to identify the facilitators for accessing evidence-based interventions for IBD-PD to inform the design and implementation of patient-centered models for IBD mental health support in the future.

**Methods:**

This was a qualitative research study in which a semi-structured interview script was developed by a multi-disciplinary team guided by the domains of the COM-B Behaviour Change Wheel framework. Participants were asked about facilitators and preferences related to psychological support services throughout their disease course. Using thematic analysis, codes were generated to identify themes using an inductive approach.

**Results:**

Fourteen participants were successfully recruited (n=14). Thematic analyses identified the following major themes: 1) Mental health should be treated as an integrated component of specialty IBD care; 2) Use of self-help strategies alongside existing supports is feasible, acceptable, and accessible; 3) Accessing support for IBD-PD through virtual care is often acceptable; 4) Flexible, multifaceted delivery models for IBD-PD are needed. All participants felt that mental health should be discussed at IBD clinic visits. Preferences for a range of psychological support formats were clear. Half of the participants said that they did not access formal psychological support services. Instead, this group preferred informal support networks or self-help strategies they already had in place. Most participants felt it was important for psychological support persons to also have IBD knowledge, but there were concerns regarding access to these services. Overall, participants felt strongly that a more qualified psychologist, even in the absence of IBD knowledge, was their top priority**.** No single approach or setting seemed to be acceptable for everyone. This illustrated that offering hybrid formats for IBD care would provide the greatest benefit to patients.

**Conclusions:**

Facilitators to accessing care start with the IBD healthcare provider, but it is important to recognize that other facilitators such as informal support through family and friends can also have an impact on care. Structural health system changes that support the delivery of integrated care still need to occur to improve overall access to multidisciplinary care.

**Funding Agencies:**

None

